# Forecasting Trends in Androgen Deprivation Therapy Intensification for Metastatic Hormone-Sensitive Prostate Cancer: A Retrospective Population-Based Cohort and Time-Series Analysis

**DOI:** 10.3390/curroncol33050276

**Published:** 2026-05-08

**Authors:** Ealia Khosh Kish, Erind Dvorani, Refik Saskin, Andrew S. Wilton, Raj Satkunasivam, Khatereh Aminoltejari, Amanda Hird, Kasey Berscheid, Soumyajit Roy, Scott C. Morgan, Michael Ong, Di Maria Jiang, Geoffrey T. Gotto, Bobby Shayegan, Girish S. Kulkarni, Rodney H. Breau, Aly-Khan A. Lalani, David-Dan Nguyen, Christopher J. D. Wallis

**Affiliations:** 1 Faculty of Medicine, University of Toronto, Toronto, ON M5S 1A1, Canada; ealia.khoshkish@mail.utoronto.ca; 2 ICES, Toronto, ON M4N 3M5, Canada; erind.dvorani@ices.on.ca (E.D.); refik.saskin@ices.on.ca (R.S.); drew.wilton@ices.on.ca (A.S.W.); 3 Institute of Health Policy, Management and Evaluation, Dalla Lana School of Public Health, University of Toronto, Toronto, ON M5S 1A1, Canada; 4 Department of Urology, Houston Methodist Hospital, Houston, TX 77030, USA; rsatkunasivam@houstonmethodist.org; 5 Division of Urology, Department of Surgery, University of Toronto, Toronto, ON M5S 1A1, Canada; kaminoltejari@hrh.ca (K.A.); amanda.hird@sunnybrook.ca (A.H.); kasey.berscheid@uhn.ca (K.B.); girish.kulkarni@uhn.ca (G.S.K.); 6 Division of Urology, Department of Surgery, Princess Margaret Cancer Centre, University Health Network, Toronto, ON M5G 2C4, Canada; 7 Division of Urology, Department of Surgery, Sunnybrook Health Sciences, University of Toronto, Toronto, ON M5S 1A1, Canada; 8 Department of Radiation Oncology, University Hospitals Seidman Cancer Center, Case Western Reserve University, Cleveland, OH 44106, USA; soumyajit.roy2@uhhospitals.org; 9 Department of Radiology, Radiation Oncology and Medical Physics, The Ottawa Hospital Cancer Centre, University of Ottawa, Ottawa, ON K1G 5Z3, Canada; smorgan@toh.ca; 10 Division of Medical Oncology, Department of Medicine, The Ottawa Hospital, Ottawa, ON K1H 1C4, Canada; mong@toh.ca; 11 Department of Medical Oncology, Princess Margaret Cancer Centre, University of Toronto, Toronto, ON M5S 1A1, Canada; di.jiang@uhn.ca; 12 Departments of Surgery and Oncology, University of Calgary, Calgary, AB T2N 1N4, Canada; 13 Division of Urology, Department of Surgery, St. Joseph’s Healthcare Hamilton, McMaster University, Hamilton, ON L8V 5C2, Canada; shayeb@mcmaster.ca; 14 Division of Urology, Department of Surgery, The Ottawa Hospital Research Institute, University of Ottawa, Ottawa, ON K1G 5Z3, Canada; rbreau@toh.ca; 15 Department of Medical Oncology, Juravinski Cancer Centre, McMaster University, Hamilton, ON L8V 5C2, Canada; lalania@hhsc.ca; 16 Division of Urology, Department of Surgery, Mount Sinai Hospital, University of Toronto, Toronto, ON M5S 1A1, Canada

**Keywords:** androgen deprivation therapy, treatment intensification, metastatic hormone-sensitive prostate cancer, ARIMA forecasting, population-based analysis, health services research, Canada

## Abstract

Treatment intensification with androgen receptor pathway inhibitors (ARPIs) and/or docetaxel in addition to androgen deprivation therapy (ADT) improves survival for men with metastatic hormone-sensitive prostate cancer (mHSPC), yet real-world uptake has historically been low. We conducted a population-based retrospective cohort study of Ontario men aged ≥66 years diagnosed with de novo mHSPC between 2014 and 2022 using linked administrative health data, defining treatment intensification as initiation of an ARPI and/or docetaxel with ADT within six months of diagnosis. Quarterly intensification rates were modeled using autoregressive integrated moving average (ARIMA) time-series methods with nonlinear trend specifications, and competing models were compared using information criteria, out-of-sample hold-out forecast accuracy, and long-horizon extrapolation behaviour to project uptake through 2030. Among 6099 men, 24% received treatment intensification, with quarterly intensification rates increasing from 3% in 2014 to 56% in 2022. A restricted cubic spline ARIMA model (ARIMA(1,0,1) + RCS3) was selected as the primary base-case forecast because it showed superior out-of-sample hold-out accuracy and more tempered long-horizon extrapolation. The cubic specification was retained as an upper-bound scenario, reflecting the possibility of continued aggressive momentum in treatment adoption. Both specifications captured a marked inflection after 2020 that temporally coincided with guideline updates and funding expansions. Near-term base-case projections (through 2026) suggest continued growth in intensification toward 80–85%, with the upper-bound scenario approaching saturation more quickly. Projections beyond 2026 are exploratory and presented for methodological completeness, given the eight-year horizon relative to a nine-year observation window and the widening prediction intervals at extended horizons. Despite substantial growth over time, treatment intensification remains incomplete in routine practice. These findings are temporally consistent with the impact of policy and funding changes on the adoption of evidence-based therapy and underscore the need for ongoing implementation efforts to address persistent clinical and system-level barriers to equitable access.

## Simple Summary

Modern treatments added to standard hormone therapy can help men with prostate cancer that has already spread live longer, but many patients do not receive these stronger treatments in everyday care. Using health system data from Ontario, we examined how often these treatments have been used over the past decade and estimated how their use may change in the future. We found that use of these more effective treatment combinations has increased substantially over time, particularly after changes in treatment guidelines and public funding, but many patients are still not receiving them. Our projections suggest that use will likely continue to rise over the next several years, though it may not reach all eligible patients. These findings can help health systems plan for future treatment needs, identify gaps in care, and guide efforts to ensure that patients have fair access to life-prolonging therapies.

## 1. Introduction

Over the past decade, treatment-intensification strategies that add androgen receptor pathway inhibitors (ARPIs) and/or docetaxel to androgen-deprivation therapy (ADT) have been shown to provide substantial survival benefits for patients with metastatic hormone-sensitive prostate cancer (mHSPC). Despite this, real-world utilization remains low, with population-based studies indicating that only about one-third of eligible men receive intensified therapy [1,2,3,4,5]. Randomized trials such as CHAARTED, LATITUDE, ENZAMET, TITAN, and ARASENS have demonstrated that ARPIs (abiraterone, enzalutamide, apalutamide, and darolutamide) and docetaxel each reduce the risk of death by approximately 25–38% compared with ADT alone [6,7,8,9,10,11,12,13].

Older age, comorbidity burden, and management by physicians other than medical oncologist are associated with lower odds of receiving intensified therapy [1,14,15]. However, little is known about how adoption is changing in response to evolving policies, expanded funding, and shifts in clinical practice. While prior Canadian population-based studies have characterized real-world intensification rates and identified predictors of receipt, none have applied explicit time-series forecasting to project future adoption trajectories or to quantify the temporal association between policy implementation and observed trend inflections. The present study extends this body of evidence with an updated observation window through 2022 and a structured multi-model forecasting framework.

This study quantifies future intensification patterns to inform policy, guide resource planning, and support educational initiatives that promote equitable delivery of life-prolonging therapy for all patients with mHSPC. We applied an autoregressive integrated moving-average (ARIMA) population-level analysis to model historical and projected trends in treatment intensification among men with mHSPC in Ontario, Canada.

## 2. Materials and Methods

### 2.1. Data Sources and Study Cohort

We identified men with mHSPC from the Ontario Cancer Registry, linking to administrative data from the Registered Persons Database, Ontario/National Drug Benefit, and Activity Level Reporting. We defined treatment intensification as the addition of an ARPI and/or docetaxel with an LHRH agonist within six months of diagnosis.

The cohort included men aged ≥66 years diagnosed with mHSPC between 1 January 2014 and 31 December 2022, with Ontario Health Insurance Plan (OHIP) coverage. De novo mHSPC was defined as stage IV prostate cancer at first diagnosis with no prior prostate cancer registration in the Ontario Cancer Registry; stage IV status was operationalized using the TNM staging field in the OCR with topography code C61. ARPIs (abiraterone acetate, enzalutamide, apalutamide, or darolutamide) and docetaxel were identified through Ontario Drug Benefit claims and Activity Level Reporting and were attributed as intensification if dispensed within six months of the index diagnosis date during a period of concurrent LHRH agonist use. Quarterly denominators were constructed as the count of newly diagnosed patients who initiated ADT in each calendar quarter, with each patient contributing to exactly one quarter based on their ADT start date. All included patients had synchronous (de novo) metastatic disease by design, as cohort eligibility required stage IV classification at first diagnosis. Patients with metachronous metastatic disease were not eligible and were not included in this cohort.

We excluded patients with continuous prednisone use (5–10 mg once daily for ≥3 months with ≤14-day gaps between prescriptions) or a rheumatology visit in the prior year, as abiraterone acetate requires concurrent low-dose prednisone, which cannot be reliably distinguished from prednisone prescribed for inflammatory conditions in administrative data. Furthermore, rheumatology visits served as a validated proxy for underlying inflammatory disease. This exclusion was applied to reduce outcome misclassification and affected a small proportion of the otherwise eligible cohort. This prednisone-based approach to identifying abiraterone exposure in ICES data has been previously described and validated [14]. They were also excluded if ADT was not initiated within 60 days before or 6 months after diagnosis. Data were aggregated quarterly.

### 2.2. Statistical Analysis

Six ARIMA models with varying trend specifications (cubic, RCS3, and piecewise with a 2020 Q1 breakpoint, each under ARIMA(1,0,1) and ARIMA(2,0,1) structures) were compared using in-sample information criteria (AIC and BIC), out-of-sample hold-out forecast accuracy, and long-horizon extrapolation behaviour; full model specifications are provided in Appendix A. The restricted cubic spline specification (ARIMA(1,0,1) + RCS3) was designated as the primary, base-case forecasting model on the basis of its substantially superior out-of-sample hold-out performance (RMSE 0.026 vs. 0.22–0.25 for cubic specifications, an approximately ten-fold difference) and its more tempered long-horizon extrapolation. The cubic specification (ARIMA(1,0,1) + cubic), although yielding the lowest in-sample AIC/BIC, is reported as an upper-bound scenario reflecting the trajectory under continued aggressive momentum in adoption, recognising that this specification saturates rapidly and is more sensitive to recent data points. Although the four-quarter hold-out window coincided with a phase of pronounced acceleration in intensification rates, this structural shift may have penalized models that extrapolated recent momentum more aggressively. However, the magnitude of the cubic model’s hold-out error and its rapid tail saturation support the more tempered RCS3 specification as the most credible base-case for long-term projection. Missing data for key variables including stage, PSA level, and physician specialty designation were minimal (<5%). For the descriptive analyses in Table 1, missing values for categorical covariates with low missingness (<1% for rurality, income quintile, and Local Health Integration Network (LHIN); <0.8% for the Ontario Marginalization Index quintiles) are displayed as a separate row in the table for full transparency on data completeness, rather than being dropped from the denominator. Given the negligible level of missingness, complete-case re-denomination would change the reported proportions by less than 0.3 percentage points across all categories, an amount smaller than the one-decimal-place rounding of the table and would not affect any descriptive comparison or substantive interpretation. This approach does not affect the time-series modeling, which operates on aggregate quarterly rates. Quarterly aggregation was selected to balance temporal resolution with statistical stability; however, this approach may obscure intra-quarter variation and provider-level heterogeneity in prescribing patterns, which should be considered when interpreting the data.

## 3. Results

### 3.1. Study Population

**Figure 1:**
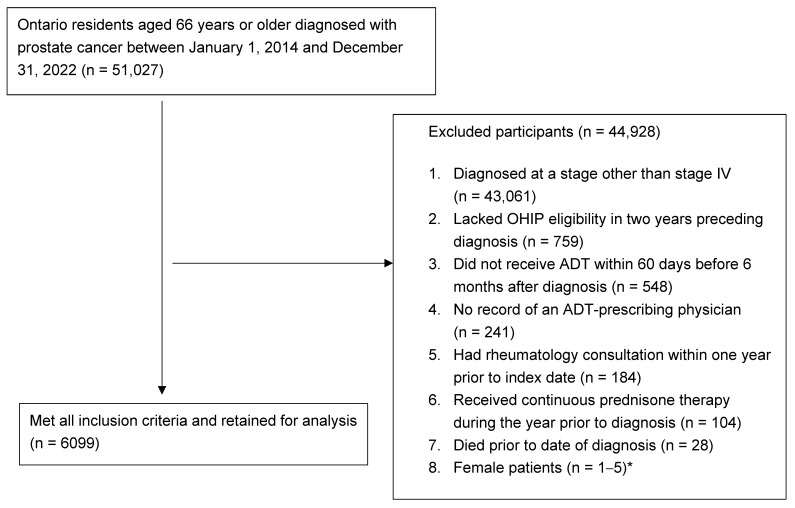
Patient selection flow diagram. The diagram outlines the construction of the analytic cohort, beginning with all Ontario men aged 66 years and older with a new diagnosis of de novo metastatic hormone-sensitive prostate cancer (mHSPC) identified between 1 January 2014 and 31 December 2022 in the Ontario Cancer Registry, and applying sequential exclusion criteria to arrive at the final analytic cohort of 6099 patients used in the time-series analysis. * Exact cell value is masked according to ICES privacy policy to prevent identification of individuals.

A total of 6099 Ontario men aged 66 years and older diagnosed with de novo mHSPC were included in this study (Figure 1). The mean age at diagnosis was 77.5 years (SD 7.5), median Charlson Comorbidity Index of 1 (IQR 0–2), and a median prostate-specific antigen (PSA) level of 74 ng/mL (IQR 0–331; maximum 16,312 ng/mL). Urologists initiated ADT for most patients (68.7%). Overall, 1475 (24%) received treatment intensification: 63.3% with ARPI alone, 32.7% with docetaxel alone, and 4.0% with both. This overall proportion reflects cumulative intensification across the full study period using all 6099 eligible patients as the denominator. Quarterly intensification rates increased from 3% in 2014 Q1 to 56% in 2022 Q4 and represent the proportion of newly ADT-initiating patients in each calendar quarter who received combination therapy within six months. These quarterly proportions capture temporal changes in prescribing practice rather than a cumulative prevalence estimate. Baseline sociodemographic and clinical characteristics of the cohort, stratified by receipt of treatment intensification, are summarized in Table 1. In brief, patients receiving intensified therapy were younger (mean age 75.0 vs. 78.2 years), had lower comorbidity burden (Charlson category 3+: 2.9% vs. 4.7%), and were more likely to be managed by medical oncology (9.4% vs. 5.8%) than those who did not receive intensification. Rates of diabetes, hypertension, COPD, and renal disease were higher among patients not receiving intensification, consistent with greater frailty in the non-intensified group.

### 3.2. Observed and Model Trends

**Figure 2:**
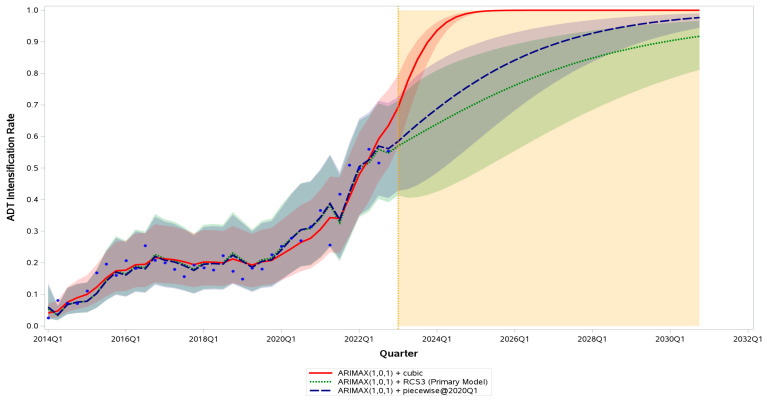
Observed and forecasted quarterly rates of ADT intensification among Ontario men aged ≥66 years with de novo metastatic hormone-sensitive prostate cancer (mHSPC), 2014Q1–2030Q4. Blue dots represent observed quarterly intensification rates. Three representative autoregressive integrated moving-average (ARIMA) trend specifications are shown: ARIMA(1,0,1) + RCS3 (green dotted line; primary, base-case model), ARIMA(1,0,1) + cubic (red solid line; upper-bound scenario reflecting continued aggressive momentum in adoption), and ARIMA(1,0,1) + piecewise@2020Q1 (blue dashed line; intermediate scenario). Shaded bands around each curve represent the corresponding 95% prediction intervals. The vertical orange dotted line marks the end of the observed series (2022 Q4) and the start of the forecast horizon; the orange-shaded region indicates the forecast period, with projections beyond 2026 interpreted as exploratory and presented for methodological completeness.

Between 2014 Q1 and 2022 Q4, the quarterly rate of treatment intensification in Ontario increased from 3% to 56% (Figure 2). Across models, ARIMA(1,0,1) + cubic produced the lowest AIC (15.42) and BIC (24.92), followed by ARIMA(2,0,1) + cubic (AIC = 17.39; BIC = 28.48). Among non-cubic trends, ARIMA(1,0,1) + piecewise@2020Q1 achieved AIC = 26.00 and BIC = 33.92, while ARIMA(2,0,1) + piecewise@2020Q1 yielded AIC = 27.88 and BIC = 37.38.

### 3.3. Projections

Assuming continuation of current trends, time-series models suggest that intensification rates may continue to rise through the near-term forecast horizon. Under the primary, base-case ARIMA(1,0,1) + RCS3 model, intensification is projected to approach 80–85% by 2026 and 90% by 2030 Q1. The cubic specifications, presented as an upper-bound scenario reflecting continued aggressive momentum in adoption, project rates approaching or exceeding 95% by 2024 Q2. The piecewise ARIMA specifications produce an intermediate trajectory, projecting a steady rise from 2023 Q1 and approaching 95% by 2029 Q2. The considerable divergence between these trajectories, which span more than six years in the timing of the 90% threshold, reflects the sensitivity of long-horizon forecasts to model specification. Projections beyond 2026 should be interpreted as exploratory and presented for methodological completeness; the relatively short observation window (nine years) cannot reliably support deterministic prediction across the full eight-year forecast horizon, and prediction intervals widen substantially after 2026. It also underscores that all projections represent conditional scenario estimates under differing trend assumptions, rather than deterministic predictions of future uptake. The observed inflection between 2019 Q4 and 2020 Q2 corresponded to the slope change modeled in the piecewise and interior knot of the RCS specifications. All models demonstrated adequate visual fit to 2014–2022 data, with residuals centered around zero and no serial autocorrelation. Formal augmented Dickey–Fuller testing of the logit-transformed intensification series supported stationarity at the zero-mean specification (lag 0, *p* = 0.003), and stationarity was consistently achieved after first differencing for models requiring it (*p* < 0.001). Residual autocorrelation diagnostics for the primary ARIMA(1,0,1) + RCS3 model confirmed absence of significant residual autocorrelation across all tested lags (Ljung–Box Q: lag 6, *p* = 0.94; lag 12, *p* = 0.49; lag 18, *p* = 0.86; lag 24, *p* = 0.71). Sensitivity analyses evaluating alternative breakpoints at 2019 Q4, 2020 Q2, 2021 Q3, 2021 Q4, and 2022 Q1 produced essentially equivalent model fit to the primary 2020 Q1 specification (ΔAIC and ΔBIC < 0.3 across all tested breakpoints), well below commonly accepted thresholds for meaningful model improvement, and yielded nearly superimposed fitted trajectories. These results support the robustness of the 2020 Q1 breakpoint to reasonable alternative choices.

Out-of-sample hold-out validation, using the final four observed quarters as the test set, further supported the restricted cubic spline (RCS3) specifications as having the lowest forecast error (ARIMA(2,0,1) + RCS3: RMSE 0.024, MAE 0.018; ARIMA(1,0,1) + RCS3: RMSE 0.026, MAE 0.019), followed by the piecewise specifications (RMSE approximately 0.04), while cubic specifications demonstrated the largest hold-out error (RMSE 0.22–0.25), reflecting the sensitivity of higher-order polynomial extrapolation to recent data. Together with the cubic specifications’ rapid tail saturation and resulting clinically implausible early plateau, this approximately ten-fold difference in hold-out error supported designating the ARIMA(1,0,1) + RCS3 specification as the primary, base-case forecasting model and reframing the cubic specification as an upper-bound scenario reflecting continued aggressive momentum in adoption. All six model forecasts through 2030 Q4 are presented with 95% prediction intervals (Figure 2; Appendix A), which widen substantially at longer horizons, consistent with the increasing uncertainty inherent to extended projections. Stratified time-series analyses by modality identified distinct adoption trajectories. Among the ARPI subgroup, the ARIMA(1,0,1) + cubic model achieved the best fit (AIC 43.4; hold-out RMSE 0.029). Among the docetaxel subgroup, the trajectory followed a long, low baseline followed by a marked post-2020 increase; piecewise specifications with active AR/MA structure produced unstable long-run forecasts, and an ARIMAX(0,0,1) + cubic model provided the most stable fit (AIC 34.4; hold-out RMSE 0.020).

## 4. Discussion

In this study, quarterly ADT intensification among men with mHSPC rose from 3% in 2014 to 56% in 2022. This increase coincided temporally with several major policy and guideline changes, including the COVID-19 state-of-emergency declaration in March 2020, the 2021 Ontario Health guideline update endorsing ADT intensification with docetaxel or ARPIs, and CADTH pERC decisions enabling publicly funded ARPI access, among other concurrent developments [16]. Although the timing of the observed inflection around 2020 Q1 aligns with these events, our study design cannot establish causation. The observed slope change may reflect the compounding effects of multiple simultaneous events, secular shifts in clinical culture, or other unobserved factors. An interrupted time-series analysis with pre-specified intervention points would be required to assess the independent contribution of individual policy changes.

Despite rising uptake, a substantial share of men with mHSPC do not receive intensified therapy. Only about one-third of eligible men receive treatment intensification despite clear survival benefits [1,14]. In Alberta, just 42% of mHSPC received intensified therapy, with older age and recurrent presentation independently predicting lower use [17]. These findings parallel those in Ontario, underscoring persistent gaps in uptake even within universal healthcare systems. A share of this gap reflects patients for whom combination therapy is clinically inappropriate, including those with frailty, significant comorbidities, drug-specific contraindications, or an informed preference to avoid treatment intensification. A second share represents eligible patients who would benefit from combination therapy but do not receive it, while a third share reflects patients whose treatment is incompletely captured by administrative data, such as privately funded therapy or care delivered outside the linked data environment. The reported gap therefore should not be interpreted uniformly as undertreatment. The therapeutic landscape for mHSPC evolved substantially over this period: prior to 2015, ADT monotherapy represented the standard of care, after which the CHAARTED and STAMPEDE trials established docetaxel as the first combination strategy to demonstrate an overall survival benefit [8,18]. Subsequent evidence from LATITUDE, ENZAMET, TITAN, ARCHES, and ARASENS progressively validated ARPI-based intensification across disease volumes and risk strata, with reductions in mortality of 25–38% over ADT alone [4,5,6,7,19]. These sequential evidence milestones were translated into guideline endorsements and public funding decisions across different timeframes in Ontario, which likely explains the stepwise pattern of adoption observed in the present data. Clinician barriers include concerns about tolerability in older or comorbid patients, inertia in escalating therapy, and specialty-based comfort differences [1]. System barriers include delayed ARPI funding, limited oncology access, and fragmented referral pathways. Patient-level barriers include hesitancy related to perceived treatment toxicities [18,19]. Strengthening interdisciplinary collaboration and administrative infrastructure may help close these gaps and improve adoption [18].

The aggregate provincial uptake trends observed in this study may not be uniformly distributed across the Ontario population. A companion population-based analysis of the same cohort demonstrated that greater area-level sociodemographic marginalization was independently associated with lower odds of receiving combination therapy (OR 0.91, 95% CI 0.83–0.99), with the greatest disparity observed in areas with higher concentrations of racialized and newcomer populations [19]. Rural residents were also significantly less likely to receive combination therapy than those residing in high-income urban areas. These disparities persisted within a universal, publicly funded healthcare system where drug costs are not a primary barrier, underscoring that financial coverage alone is insufficient to ensure equitable access [20,21]. Structural factors such as limited geographic access to medical oncology, reduced availability of culturally safe navigation and support services, and social circumstances that impede patients’ ability to attend specialist visits or tolerate the logistical demands of combination therapy further contribute to these inequities [18,22]. Critically, greater marginalization was also associated with reduced referral to medical oncology, the specialty most strongly associated with treatment intensification, suggesting that differential specialist access represents a key mediating pathway [23]. These findings highlight that aggregate intensification trends may mask meaningful within-population disparities requiring targeted, equity-driven interventions.

Closing these persistent gaps will require coordinated, system-level strategies that extend well beyond guideline dissemination. In a large and geographically diverse province like Ontario, multidisciplinary models of care are essential. These include integrated urology-oncology clinics, rapid-access consultation pathways, and structured tumor board processes to ensure that all patients, regardless of the specialty that first prescribes their ADT or where they live, receive timely specialist evaluation and an informed intensification discussion [24,25]. Structured referral protocols and patient navigation programs represent modifiable system levers to reduce practice variation driven by prescriber specialty, geographic distance, and sociodemographic disadvantage [22]. Equity-informed implementation strategies such as mobile or remote oncology consultation services for rural and northern Ontario communities, culturally appropriate patient education, and proactive registry-level identification of patients who have not yet received intensification are particularly important given the structural disparities documented in this province. The projected uptake gains modeled in this study will translate into equitable population-level benefit only if system investments actively account for and mitigate these structural barriers.

The restricted cubic spline (RCS3) ARIMA model was selected as the primary, base-case forecast given its superior out-of-sample hold-out performance and more tempered long-horizon extrapolation, capturing a smooth nonlinear rise in ADT intensification, rapid early growth, and gradual recent plateauing. The cubic specification, although it produced the lowest in-sample AIC/BIC, is reported as an upper-bound scenario reflecting continued aggressive momentum in adoption (e.g., further guideline endorsement, expanded ARPI availability), recognising that this specification saturates rapidly and is more sensitive to recent observations. Under the base-case RCS3 model, intensification is projected to approach 80–85% by 2026 and 90% by 2030 Q1, while the upper-bound cubic scenario projects rates approaching 95% by 2024 Q2. Both should be interpreted as model-dependent scenarios rather than expected outcomes, and projections beyond 2026 are exploratory and presented for methodological completeness. In routine practice, a meaningful subset of patients will remain unsuitable for intensification due to advanced frailty, significant comorbidities contraindicating specific agents, informed patient preference against additional toxicity, or ongoing system-level access limitations [26]. These clinical realities impose an effective ceiling on achievable uptake that is likely well below 100%, and forecasts should therefore be used to establish ambitious but clinically credible benchmarks. Comparing future observed rates with these forecasts will identify residual gaps and factors limiting full adoption despite strong evidence and policy support. We emphasize that the time-series models presented here are necessarily imperfect representations of a complex, policy-driven adoption process, and are not intended as predictive models of individual patient treatment. Rather, the goal of this analysis is to use data-driven estimates to describe observed trends and to generate associated forecasts of population-level systemic therapy use. Because future uptake may be influenced by unanticipated clinical, regulatory, or health-system changes, we interpret these results as informative for forecasting under observed historical patterns, rather than as definitive predictions of future use.

This study has several limitations. First, the cohort was restricted to men aged ≥66 years because Ontario Drug Benefit data provides incomplete coverage for younger patients enrolled in private drug plans, limiting generalizability to younger men with mHSPC. Second, this is a descriptive time-series analysis: the 2020 Q1 inflection coincides temporally with guideline updates and funding expansions, but causal inference cannot be established, and long-horizon projections are inherently sensitive to model specification (as evidenced by divergence between cubic and RCS3 forecasts) and to unanticipated changes in drug funding or clinical practice. Forecasts beyond 2026 should be interpreted as exploratory rather than as deterministic projections, given the eight-year horizon relative to the nine-year observation window and the substantial widening of prediction intervals at extended horizons. All projections should therefore be interpreted as conditional scenarios. Third, administrative data may not fully capture privately funded therapies, particularly in earlier years of the study period when abiraterone access relied on compassionate use programs or private insurance. These data also do not permit reliable identification of patients clinically ineligible for intensification due to frailty or comorbidity and do not include disease volume per CHAARTED criteria; therefore, the reported treatment gap should not be interpreted uniformly as undertreatment. Fourth, exclusion of patients with chronic prednisone use or a recent rheumatology encounter, applied to reduce misclassification of abiraterone-related from non-oncologic prednisone use, may have removed a small subgroup that is, on average, older and frailer than retained patients. Because such patients are also less likely to receive intensification, this exclusion could plausibly bias the estimated intensification rate modestly upward. The proportion of otherwise eligible patients excluded by this criterion is small (reported in Figure 1), and the same operational definition has been validated in prior population-based work using the same data environment [14]. Nevertheless, the directionality of any residual bias should be considered when interpreting the absolute level of intensification, although the strong temporal trend (3% to 56%) is unlikely to be materially affected. Fifth, evolving diagnostic methods for staging metastatic disease, including the introduction of PSMA PET-CT imaging in more recent years, may have influenced both the identification of metastatic disease and the apparent trend in treatment decisions [27,28,29,30]. Finally, quarterly aggregation may obscure provider-level heterogeneity and intra-quarter variation in prescribing, and the inclusion of pre-intensification era data (when combination therapy was not yet endorsed or publicly funded) introduces some temporal heterogeneity at the start of the observation window.

## 5. Conclusions

In this population-based cohort study of older men with de novo mHSPC in Ontario, quarterly ADT intensification rates rose substantially from 3% in 2014 to 56% in 2022, with a notable inflection temporally coinciding with major guideline updates and public funding expansions around 2020. Time-series forecasting using a restricted cubic spline ARIMA model as the primary, base-case forecast projects continued near-term growth, with intensification approaching 80–85% by 2026 and 90% by 2030 Q1; an upper-bound cubic scenario projects faster saturation. Given the eight-year horizon relative to the nine-year observation window and the substantial widening of prediction intervals at extended horizons, projections beyond 2026 are exploratory and presented for methodological completeness. These projections represent model-dependent scenarios rather than expected realized uptake; frailty, contraindications, informed patient preference, and structural barriers will likely constrain achievable uptake below the modelled ceilings. These findings provide health system planners with population-level estimates to anticipate future demand, guide resource allocation, and target implementation efforts. Ongoing surveillance of real-world intensification rates against these projected trajectories, stratified by geography, prescriber specialty, and sociodemographic status, is essential to ensure that the survival benefits of combination therapy are realized equitably across all patients with mHSPC.

## Figures and Tables

**Table 1:** Baseline demographic, clinical, geographic, and neighbourhood-level characteristics of the complete cohort, stratified by receipt of ADT treatment intensification within 6 months of diagnosis.

Variable_Label	Variable_Value	Total_Cohort	No_Intensified_Tx	Intensified_Tx	StdDiff	*p*_Value
ADT Intensification Treatment with any Drug Within 6 Months of Diagnosis	Sample Size	N = 6099	N = 4624	N = 1475		
Physician main specialty group	GP/FP	354 (5.80%)	284 (6.14%)	70 (4.75%)	0.062	<0.0001
	Med Onc	405 (6.64%)	266 (5.75%)	139 (9.42%)	0.139	
	Other Spec	265 (4.34%)	202 (4.37%)	63 (4.27%)	0.005	
	Rad Onc	884 (14.49%)	728 (15.74%)	156 (10.58%)	0.153	
	Urologist	4191 (68.72%)	3144 (67.99%)	1047 (70.98%)	0.065	
Age at index date	Mean (SD)	77.47 (7.35)	78.24 (7.48)	75.04 (6.36)	0.461	<0.0001
	Median (Q1–Q3)	77 (71–83)	78 (72–84)	74 (70–79)	0.447	<0.0001
	Missing (%)	0.0%	0.0%	0.0%		
	Min–Max	66–100	66–100	66–95		
Year of PCa diagnosis	2014	492 (8.07%)	461 (9.97%)	31 (2.10%)	0.335	<0.0001
	2015	587 (9.62%)	492 (10.64%)	95 (6.44%)	0.151	
	2016	605 (9.92%)	478 (10.34%)	127 (8.61%)	0.059	
	2017	641 (10.51%)	524 (11.33%)	117 (7.93%)	0.115	
	2018	798 (13.08%)	646 (13.97%)	152 (10.31%)	0.112	
	2019	874 (14.33%)	713 (15.42%)	161 (10.92%)	0.134	
	2020	810 (13.28%)	582 (12.59%)	228 (15.46%)	0.083	
	2021	830 (13.61%)	512 (11.07%)	318 (21.56%)	0.287	
	2022	462 (7.58%)	216 (4.67%)	246 (16.68%)	0.396	
Charlson category	0	632 (10.36%)	502 (10.86%)	130 (8.81%)	0.069	<0.0001
	1	300 (4.92%)	244 (5.28%)	56 (3.80%)	0.071	
	2	230 (3.77%)	183 (3.96%)	47 (3.19%)	0.042	
	3+	259 (4.25%)	216 (4.67%)	43 (2.92%)	0.092	
	M	4678 (76.70%)	3479 (75.24%)	1199 (81.29%)	0.147	
Diabetes	1	1943 (31.86%)	1477 (31.94%)	466 (31.59%)	0.007	0.8023
CHF	1	839 (13.76%)	699 (15.12%)	140 (9.49%)	0.172	<0.0001
COPD	1	1388 (22.76%)	1089 (23.55%)	299 (20.27%)	0.079	0.0089
Hypertension	1	4423 (72.52%)	3419 (73.94%)	1004 (68.07%)	0.13	<0.0001
Dementia in 5 years prior to index	1	240 (3.94%)	216 (4.67%)	24 (1.63%)	0.175	<0.0001
Myocardial infarction in 5 y prior	1	148 (2.43%)	114 (2.47%)	34 (2.31%)	0.011	0.7275
Cerebrovascular accident in 5 y prior	1	120 (1.97%)	109 (2.36%)	11 (0.75%)	0.131	0.0001
Arrhythmia in 1 y prior	1	85 (1.39%)	70 (1.51%)	15 (1.02%)	0.044	0.1564
Liver Disease in 5 y prior	1	48 (0.79%)	36 (0.78%)	12 (0.81%)	0.004	0.8946
Renal Disease in 5 y prior	1	359 (5.89%)	292 (6.31%)	67 (4.54%)	0.078	0.0118
Rurality	Missing	14 (0.23%)	*9–13	*1–5	0.011	0.8425
	N	5178 (84.90%)	3921 (84.80%)	1257 (85.22%)	0.012	
	Y	907 (14.87%)	*690–694	*213–217	0.013	
Income Quintile	Missing	19 (0.31%)	*14–18	*1–5	0.01	0.257
	1	1222 (20.04%)	947 (20.48%)	275 (18.64%)	0.046	
	2	1224 (20.07%)	946 (20.46%)	278 (18.85%)	0.041	
	3	1201 (19.69%)	903 (19.53%)	298 (20.20%)	0.017	
	4	1143 (18.74%)	*857–861	*282–286	0.015	
	5	1290 (21.15%)	953 (20.61%)	337 (22.85%)	0.054	
LHIN Name	Missing	*1–5	*1–5	0 (0.00%)	0.021	0.0037
	Central	661 (10.84%)	505 (10.92%)	156 (10.58%)	0.011	
	Central East	569 (9.33%)	410 (8.87%)	159 (10.78%)	0.064	
	Central West	297 (4.87%)	208 (4.50%)	89 (6.03%)	0.069	
	Champlain	537 (8.80%)	422 (9.13%)	115 (7.80%)	0.048	
	Erie St. Clair	350 (5.74%)	275 (5.95%)	75 (5.08%)	0.038	
	Hamilton Niagara Haldimand Brant	765 (12.54%)	597 (12.91%)	168 (11.39%)	0.047	
	Mississauga Halton	377 (6.18%)	300 (6.49%)	77 (5.22%)	0.054	
	North East	388 (6.36%)	289 (6.25%)	99 (6.71%)	0.019	
	North Simcoe Muskoka	228 (3.74%)	173 (3.74%)	55 (3.73%)	0.001	
	North West	*182–186	*126–130	56 (3.80%)	0.055	
	South East	355 (5.82%)	282 (6.10%)	73 (4.95%)	0.05	
	South West	521 (8.54%)	373 (8.07%)	148 (10.03%)	0.069	
	Toronto Central	481 (7.89%)	375 (8.11%)	106 (7.19%)	0.035	
	Waterloo Wellington	383 (6.28%)	284 (6.14%)	99 (6.71%)	0.023	
Households and Dwellings Quintile	Missing	48 (0.79%)	38 (0.82%)	10 (0.68%)	0.017	0.0494
	1	898 (14.72%)	675 (14.60%)	223 (15.12%)	0.015	
	2	1198 (19.64%)	878 (18.99%)	320 (21.69%)	0.067	
	3	1254 (20.56%)	938 (20.29%)	316 (21.42%)	0.028	
	4	1269 (20.81%)	972 (21.02%)	297 (20.14%)	0.022	
	5	1432 (23.48%)	1123 (24.29%)	309 (20.95%)	0.08	
Material Resources Quintile	Missing	48 (0.79%)	38 (0.82%)	10 (0.68%)	0.017	0.4805
	1	1314 (21.54%)	994 (21.50%)	320 (21.69%)	0.005	
	2	1295 (21.23%)	989 (21.39%)	306 (20.75%)	0.016	
	3	1193 (19.56%)	888 (19.20%)	305 (20.68%)	0.037	
	4	1154 (18.92%)	864 (18.69%)	290 (19.66%)	0.025	
	5	1095 (17.95%)	851 (18.40%)	244 (16.54%)	0.049	
Age and Labour Force Quintile	Missing	48 (0.79%)	38 (0.82%)	10 (0.68%)	0.017	0.3048
	1	801 (13.13%)	600 (12.98%)	201 (13.63%)	0.019	
	2	994 (16.30%)	749 (16.20%)	245 (16.61%)	0.011	
	3	1074 (17.61%)	797 (17.24%)	277 (18.78%)	0.04	
	4	1276 (20.92%)	960 (20.76%)	316 (21.42%)	0.016	
	5	1906 (31.25%)	1480 (32.01%)	426 (28.88%)	0.068	
Racialized and Newcomer Populations Quintile	Missing	48 (0.79%)	38 (0.82%)	10 (0.68%)	0.017	0.4495
	1	1412 (23.15%)	1066 (23.05%)	346 (23.46%)	0.01	
	2	1347 (22.09%)	999 (21.60%)	348 (23.59%)	0.048	
	3	1224 (20.07%)	924 (19.98%)	300 (20.34%)	0.009	
	4	1107 (18.15%)	857 (18.53%)	250 (16.95%)	0.041	
	5	961 (15.76%)	740 (16.00%)	221 (14.98%)	0.028	

Notes: Income Quintile is defined at the neighbourhood level using Statistics Canada census data, with Quintile 1 representing the lowest-income group and Quintile 5 the highest-income group. Ontario Marginalization (ON-Marg) Index quintiles for Households and Dwellings, Material Resources, Age and Labour Force, and Racialized and Newcomer Populations follow standard ON-Marg conventions and are oriented in the opposite direction, with Quintile 1 representing the least marginalized neighbourhoods and Quintile 5 the most marginalized. Cell counts between 1 and 5 are suppressed in accordance with ICES privacy policy and shown as ranges. Missing values are shown as separate rows for transparency. Abbreviations: ADT = androgen deprivation therapy; CHF = congestive heart failure; COPD = chronic obstructive pulmonary disease; GP/FP = general practitioner/family physician; LHIN = Local Health Integration Network; Med Onc = medical oncologist; Rad Onc = radiation oncologist; PCa = prostate cancer; SD = standard deviation. * Exact cell value is masked according to ICES privacy policy to prevent identification of individuals.

## Data Availability

The data used in this study are housed at ICES and are not publicly available due to legal and privacy restrictions.

## Appendix A

### Appendix A.1. Expanded Statistical Analysis

We generated six ARIMA models, comparing cubic, 3-knot restricted cubic spline (RCS3), and piecewise specifications with a 2020 Q1 breakpoint, each estimated under ARIMA(1,0,1) and ARIMA(2,0,1) structures. We forecasted eight years; projecting intensification rate out to the end of 2030 Q4. Continuous variables were summarized using mean and standard deviation. Models were estimated using maximum likelihood based on the Box–Jenkins ARIMA framework. We applied logit transformation to intensification rates prior to model fitting, then back-transformed predictions to the probability scale. We assessed model fit using the Akaike Information Criterion (AIC) and Bayesian Information Criterion (BIC). All analyses followed the STROBE guidelines for cohort studies [31,32].

For all six models, we specified a maximum lag of eight quarters (two years) to capture medium-term temporal dependencies in ADT intensification rates, while minimizing over-fitting. A two-year window reflects a clinically or policy-relevant timeframe within which treatment practice patterns and funding changes in Ontario would reasonably impact physicians’ treatment decisions. All models were estimated using a maximum likelihood method in SAS Enterprise Guide 8 and were based on the Box–Jenkins ARIMA methodology, expressing the time series *X_t_* with the equation [33,34]:

*ϕ_p_*(*B*)(1 − *B*)*^d^X_t_* = *θ_q_*(*B*)*a_t_*

In this equation, *B* is the backshift operator, *d* is the order of differencing, *p* and *q* are the autoregressive and moving-average orders, respectively, and *a_t_* is the random error term at time *t*. Since no repeated seasonal pattern is anticipated with ADT intensification rate, we utilized non-seasonal ARIMA models. To account for underlying clinical and policy changes, deterministic time components such as piecewise time trends and restricted cubic spline terms were incorporated as exogenous regressors within the ARIMA framework. We explored various plausible structures to capture both short-term autocorrelation and long-term secular trends, aligning with different theoretical assumptions about how intensification rates are expected to evolve over time.

All forecasting models were fit on the logit-transformed intensification rates. Model predictions were then transformed back to the probability scale to present results as interpretable proportions bounded between 0 and 1. Missing data were minimal (<5%) for key variables. Sensitivity analysis comparing ARIMA (1,0,1) and ARIMA (2,0,1) specifications yielded consistent results, supporting model robustness. Sensitivity analyses comparing alternative ARIMA specifications (e.g., (1,0,1) vs. (2,0,1)) yielded consistent forecasts.

Ontario Health (formerly Cancer Care Ontario) released their ASCO guideline for primary management of mHSPC recommendations for intensified ADT treatment with docetaxel or ARPIs (abiraterone, enzalutamide, or abiraterone) in 2021. This, along with other policy changes and visual inspection of the quarterly data plot, informed our selection of ARIMA model framework components—the piecewise and RCS models, breaks and knot points were selected both visually and in alignment with policy implementations. Specifically, the 2020 Q1 breakpoint was selected based on the coincidence of a visible inflection in the quarterly data with the March 2020 declaration of the COVID-19 state of emergency and the contemporaneous acceleration in CADTH pERC funding decisions for ARPIs. The interior knot for the RCS3 specification was similarly placed at 2020 Q1 to reflect the same structural change.

We generated the following six distinct models with varying trend forms the following:

- ARIMA (1,0,1) + cubic: A single autoregressive and moving-average term with a smooth cubic polynomial time trend as the deterministic component, capturing gradual nonlinear acceleration in intensification rates.
- ARIMA (2,0,1) + cubic: Two autoregressive terms with the same cubic polynomial time trend, accommodating longer-lag autocorrelation in the series.
- ARIMA (1,0,1) + piecewise @2020Q1: Includes a structural break at Q1 of 2020 to capture slope change in observed data, while maintaining short-term autocorrelation.
- ARIMA (2,0,1) + piecewise @2020Q1: Same structural break with added AR term to capture a structural shift and lingering autocorrelation.
- ARIMA (1,0,1) + RCS3: Fits flexible nonlinear time trend using RCS with three knots (interior knot at 2020Q1 visually/guideline-informed window), allowing for gradual curvature in rate.
- ARIMA (2,0,1) + RCS3: Same three-knot spline model with an extra autoregressive term for extended memory influence.

Observed quarterly intensification rates and corresponding model-predicted and forecasted values were plotted on a line graph to visually evaluate model fit and to compare the projected trajectories under each time trend assumption. Model fit was also evaluated using the Akaike Information Criterion (AIC) and Bayesian Information Criterion (BIC). Both measures balance model complexity against goodness of fit, with lower values indicating a more parsimonious and better-fitting model. The models were compared based on their AIC and BIC statistics, with the lowest values considered to provide the best fit to the observed data.

To assess model adequacy and robustness, stationarity was evaluated on the transformed series using visual inspection and augmented Dickey–Fuller (ADF) testing. For non-differenced models, stationarity was assessed on the logit-transformed intensification series; for models requiring first differencing, stationarity was assessed on the first-differenced logit series. Residual autocorrelation was evaluated using residual autocorrelation function (ACF) and partial autocorrelation function (PACF) plots and by examining whether residual correlations remained within approximate 95% confidence limits. To supplement model selection based on AIC, BIC, and visual fit, we performed an out-of-sample hold-out validation by withholding the final four observed quarters, refitting each candidate model using the preceding quarters only, generating forecasts for the hold-out period, back-transforming predictions to the probability scale, and calculating forecast accuracy using root mean squared error (RMSE) and mean absolute error (MAE). We additionally evaluated alternative breakpoint specifications for the piecewise models (2019 Q4, 2020 Q2, 2021 Q3, 2021 Q4, and 2022 Q1, in addition to the primary 2020 Q1 specification), comparing model fit using AIC and BIC. Forecasts for all six models are presented with 95% prediction intervals. To complement the aggregated analysis, stratified time-series models were additionally estimated separately for intensification with ARPIs (enzalutamide, apalutamide, or abiraterone/prednisone proxy) and with docetaxel, using the same diagnosis-quarter denominator and the same modeling framework.

Efforts to minimize bias included the use of comprehensive, population-level administrative databases that capture all provincially funded prescriptions and cancer diagnoses, reducing recall and selection bias. However, potential misclassification bias may remain due to incomplete capture of privately funded medication or missing stage data. This study followed the Strengthening the Reporting of Observational Studies in Epidemiology (STROBE) reporting guidelines for cohort studies [31].

Identification of abiraterone exposure using Ontario Drug Benefit (ODB) claims alone is insufficient to fully capture true use because the abiraterone product code is incompletely populated in administrative dispensing records. Because concomitant low-dose prednisone is an intrinsic and clinically required component of abiraterone therapy, prednisone dispensing was used as a pragmatic proxy to improve capture of likely abiraterone exposure, consistent with prior population-based work in the same data environment [14]. Our objective was to reduce false negatives (missed intensification) while implementing restrictions to limit false-positive classification: we excluded patients with chronic prednisone exposure patterns consistent with non-oncologic inflammatory indications, excluded those with rheumatology encounters suggestive of alternative prednisone use, and required prednisone dispensing to occur within the prespecified treatment intensification window in the context of concurrent ADT. These design features were intended to improve specificity and distinguish likely abiraterone-associated prednisone use from background corticosteroid use. The same operational definition of treatment intensification (initiation of an ARPI [abiraterone, enzalutamide, apalutamide, or darolutamide] and/or docetaxel together with an LHRH agonist within six months of diagnosis) is used consistently throughout the manuscript.

### Appendix A.2. Model Diagnostics, Validation, and Sensitivity Analyses

Stationarity assessment. Stationarity of the logit-transformed quarterly intensification series was evaluated using visual inspection of the series and of its autocorrelation function, together with the augmented Dickey–Fuller (ADF) test. Testing was performed on both the logit-transformed series (used for non-differenced ARIMA specifications) and on its first difference (used for differenced specifications). The ADF test rejected the null of a unit root for the zero-mean specification at lag 0 (*p* = 0.003), and first differencing produced *p*-values < 0.001 across lags, supporting stationarity for the residual-based ARIMA component once a flexible trend was included in the mean structure [35].

Residual autocorrelation diagnostics. For each candidate model, we inspected residual autocorrelation function (ACF) and partial autocorrelation function (PACF) plots and verified that residual correlations remained within approximate 95% confidence limits. We additionally evaluated Ljung–Box Q statistics at lags 6, 12, 18, and 24. For the primary ARIMA(1,0,1) + RCS3 model, all Ljung–Box *p*-values were well above 0.05 (lag 6, *p* = 0.94; lag 12, *p* = 0.49; lag 18, *p* = 0.86; lag 24, *p* = 0.71), indicating no meaningful residual autocorrelation. Analogous diagnostics for the remaining five specifications likewise showed no evidence of retained autocorrelation.

Out-of-sample hold-out validation and primary model selection. In addition to information-criterion-based model selection (AIC and BIC), we performed an out-of-sample hold-out validation. The final four observed quarters (2022 Q1–Q4) were withheld, each candidate model was re-estimated using only the preceding quarters, and forecasts were generated for the hold-out period. Forecasts were back-transformed from the logit to the probability scale, and forecast accuracy was summarized using root mean squared error (RMSE) and mean absolute error (MAE) between observed and predicted quarterly proportions. Among the six candidate specifications, the restricted cubic spline (RCS3) models produced the lowest hold-out error (ARIMA(2,0,1) + RCS3: RMSE 0.024, MAE 0.018; ARIMA(1,0,1) + RCS3: RMSE 0.026, MAE 0.019), followed by the piecewise specifications (RMSE approximately 0.04). Cubic specifications yielded substantially larger hold-out error (RMSE 0.22–0.25), reflecting the sensitivity of higher-order polynomial extrapolation to recent observations. Although in-sample AIC/BIC favoured the cubic specification, we designated the ARIMA(1,0,1) + RCS3 model as the primary, base-case forecast on the basis of (i) its approximately ten-fold lower hold-out forecast error and (ii) its more tempered long-horizon extrapolation behaviour. We acknowledge that the four-quarter hold-out window coincided with a phase of pronounced acceleration in intensification rates. This structural shift may have penalized models extrapolating recent momentum more aggressively, meaning that hold-out RMSE in this setting is not a clean test of forecast accuracy under a stationary regime. However, the magnitude of the difference (RMSE 0.024–0.026 for RCS3 vs. 0.22–0.25 for cubic) and the cubic specification’s clinically implausible early plateau (rates > 95% by 2024 Q2) together exceed the threshold at which an in-sample information-criterion advantage can be considered decisive. The cubic specification is therefore retained as a transparently labelled upper-bound scenario, representing a trajectory under continued aggressive momentum in adoption (e.g., further guideline endorsement, expanded ARPI availability, sustained practice change), rather than as the base-case forecast.

Prediction intervals. For all six forecasting models, point forecasts through 2030 Q4 are reported together with 95% prediction intervals. Intervals were computed on the logit scale using the standard error of prediction under each fitted ARIMA specification and then back-transformed to the probability scale. Prediction intervals widen progressively over the forecast horizon, consistent with accumulating uncertainty in extended projections, and should be read as the primary summary of forecast uncertainty. Because intervals widen substantially after 2026, projections beyond this point should be interpreted as exploratory and presented for methodological completeness rather than as deterministic predictions of future use.

Breakpoint sensitivity analysis. The primary piecewise specifications used a 2020 Q1 breakpoint, informed by the temporal alignment of ASCO guideline updates and Ontario funding decisions. To evaluate whether this choice materially influenced model performance, we refit the piecewise ARIMA models under five alternative breakpoints: 2019 Q4, 2020 Q2, 2021 Q3, 2021 Q4, and 2022 Q1. All alternative specifications yielded nearly identical AIC and BIC values to the primary 2020 Q1 specification (ΔAIC and ΔBIC < 0.3 across all tested breakpoints), well below conventional thresholds for meaningful model improvement (ΔAIC ≥ 2), and produced nearly superimposed fitted trajectories. The primary breakpoint choice is therefore not a principal driver of model fit or forecast behaviour.

Stratified time-series analyses by treatment modality. To complement the aggregated analysis and to address reviewer comments on treatment heterogeneity, we fit parallel time-series models separately for intensification with androgen receptor pathway inhibitors (ARPIs; enzalutamide, apalutamide, or abiraterone captured via the prednisone proxy) and with docetaxel, using the same diagnosis-quarter denominator and the same modeling framework (six candidate specifications per stratum with logit transformation, AIC/BIC selection, residual diagnostics, and hold-out validation). Among the ARPI stratum, an ARIMA(1,0,1) + cubic model provided the best fit (AIC 43.4; hold-out RMSE 0.029). The docetaxel stratum showed a prolonged low baseline followed by a marked post-2020 rise; piecewise specifications with active AR/MA structure produced unstable long-run forecasts driven by the limited length of the post-breakpoint segment, and an ARIMAX(0,0,1) + cubic specification provided the most stable model fit (AIC 34.4; hold-out RMSE 0.020). Stratified results should be interpreted with caution given smaller per-stratum event counts; full stratum-specific diagnostics and model tables are available on request.

Taken together, these diagnostics and sensitivity analyses indicate that model-based projections are robust to reasonable alternative specifications of the breakpoint, but that long-horizon forecast magnitudes remain sensitive to trend specification. This sensitivity is captured by the reported prediction intervals and by the divergence between the cubic and RCS3 families, and is the basis for interpreting projections as model-dependent scenarios rather than as deterministic expected outcomes.
